# Supplementary material for: T-Cell Lymphoma Clonality by Copy Number Variation Analysis of T-Cell Receptor Genes
Source: Cancers (Basel). 2021 Jan 19;13(2):340. doi: 10.3390/cancers13020340 (PMC7832336; doi:10.3390/cancers13020340)
Supplement: Supplementary file 1 [file cancers-13-00340-s001.pdf]

Supplementary Table S1. Details of WGS, TCRB targeted sequencing and PCR of sample cases

Case no.	Case ID	Tumor %	Sequencing depth	Diagnosis	TCR genes with monclonal losses	Nature of WGS MR (n=44)					PCR clonality (n=31)	Nature of PCR rearrangement		TCRB rearrangement by PCR	TCRB rearrangement by targeted sequencing (n=12)
						TCRG	TCRB (VJ)	TCRB (DJ)	TCRA	TCRD		TCRG	TCRB		
1	PB000933	13%	71X	AITL	TCRG, TCRB, TCRA	M	M	Nil	M	Nil	NA	NA	NA	NA	NA
2	426A	15%	75X	AITL	TCRG, TCRB	M	M	Nil	Nil	Nil	NA	NA	NA	NA	NA
3	z9951	18%	70X	AITL	TCRG, TCRB, TCRA	M	M	Nil	M	Nil	MC	M	Nil	Nil	NA
4	z6425	20%	65X	PTCL	TCRG, TCRA	M	Nil	Nil	M	Nil	NA	NA	NA	NA	NA
5	z3917	20%	83X	MEITL	TCRB	Nil	M	Nil	Nil	Nil	MC	M	M	Incomplete	NA
6	AITL13	20%	67X	AITL	TCRG, TCRB, TCRA	M	M	Nil	M	Nil	MC	M	B	Complete and incomplete	NA
7	193A	21%	69X	PTCL	TCRG, TCRB, TCRA, TCRD	M	M	Nil	M	M	NA	NA	NA	NA	NA
8	TR4254	21%	71X	AITL	TCRB	Nil	M	Nil	Nil	Nil	Nil	Nil	Nil	Nil	NA
9	z8343	22%	65X	PTCL	TCRG, TCRB, TCRA	M	M	Nil	B	Nil	Nil	Nil	Nil	Nil	NA
10	z5021	23%	73X	ALCL	TCRG, TCRB, TCRA	M	M	Nil	M	Nil	MC	Nil	M	Complete	NA
11	z7889	23%	74X	PTCL	TCRG, TCRB, TCRA	B	M	Nil	B	Nil	NA	NA	NA	NA	NA
12	TR4323	23%	65X	AITL	TCRG, TCRB, TCRA	B	M	Nil	B	Nil	MC	M	M	Complete	Complete
13	TR4313	23%	71X	AITL	TCRG, TCRB, TCRA	M	Nil	Yes	M	Nil	NA	NA	NA	NA	NA
14	PB30818	23%	66X	ALCL	NIL	Nil	Nil	Nil	Nil	Nil	NA	NA	NA	NA	NA
15	229A	23%	61	AITL	TCRG, TCRB, TCRA	M	M	Nil	M	Nil	MC	B	MC-IND	Complete	NA
16	PTCL02	23%	67X	PTCL	TCRG, TCRA	M	Nil #	Nil	B	Nil	MC	M	M	Incomplete	Complete and incomplete
17	z7285	23%	70X	AITL	TCRG, TCRB, TCRA	B	M	Nil	B	Nil	Nil	Nil	Nil	Nil	NA
18	T1443KC	24%	69X	AITL	TCRG, TCRB, TCRA	B	B	Nil	B	Nil	MC	B	MC-IND	Complete and incomplete	NA
19	T1482TA	25%	62X	AITL	TCRG, TCRB	M	M	Nil	Nil	Nil	NA	NA	NA	NA	NA
20	496A	26%	71X	AITL	TCRG, TCRB, TCRA	B	M	Nil	B	Nil	MC	B	B	Complete and incomplete	NA
21	TR4250	26%	70X	AITL	TCRG, TCRB, TCRA	B	B	Nil	B	Nil	NA	NA	NA	NA	NA
22	TR4241	27%	63X	AITL	TCRG, TCRB	M	M	Nil	Nil	Nil	MC	M	M	Complete	NA
23	69A	27%	68X	PTCL	TCRG, TCRB, TCRA	M	M	Nil	M	Nil	MC	B	Nil	Nil	Complete
24	211A	29%	65X	AITL	TCRG, TCRB, TCRA	M	M	Yes	M	Nil	MC	B	B	Complete and incomplete	Complete and incomplete
25	z4739	30%	61X	PTCL	TCRG, TCRB, TCRA	M	M	Yes	M	Nil	MC	B	B	Complete and incomplete	NA
26	z5286	31%	58X	ALCL	TCRG, TCRA, TCRD	B	Nil	Nil	B	M	MC	B	M	Incomplete	Incomplete
27	62A	41%	77X	PTCL	TCRG, TCRB, TCRA, TCRD	B	B	Nil	M	M	NA	NA	NA	NA	NA
28	z3706	41%	70X	MEITL	TCRG, TCRB, TCRA, TCRD	M	Nil	Yes	B	B	MC	M	M	Incomplete	Incomplete
29	TR4290	42%	71X	PTCL	TCRG, TCRB, TCRA	M	M	Nil	M	Nil	MC	B	MC-IND	Complete and incomplete	NA
30	AITL10	44%	35X	AITL	TCRG, TCRB, TCRA	B	M	Nil	B	Nil	MC	B	MC-IND	Complete and incomplete	NA
31	194A	45%	72X	AITL	TCRG, TCRB, TCRA	B	Nil	Yes	B	Nil	MC	M	M	Incomplete	Incomplete
32	SLS0697	45%	77X	HSTL	TCRG, TCRD	B	Nil	Nil	Nil	M	MC	B	IND	IND	Incomplete
33	z2756	46%	74X	PTCL	TCRG, TCRB, TCRA	B	M	Nil	B	Nil	MC	B	B	Complete and incomplete	NA
34	z8043	48%	73X	AITL	TCRG, TCRB, TCRA	B	B	Yes	B	Nil	MC	B	B	Complete	NA

35	TR4311	53%	68X	PTCL	TCRG, TCRB, TCRA	M	B	Yes	B	Nil	NA	NA	NA	NA	NA
36	z4110	69%	64X	ALCL	TCRG, TCRB, TCRA	B	B	Nil	B	Nil	MC	B	B	Complete	NA
37	116A	72%	68X	AITL	TCRG, TCRB, TCRA	B	B	Nil	B	Nil	MC	B	M	Complete and incomplete	Complete and incomplete
38	TR4232	78%	71X	AITL	TCRG, TCRB, TCRA	B	M	Nil	B	Nil	MC	M	Nil	Nil	NA
39	z3951	79%	65X	PTCL	TCRG, TCRB, TCRA	B	B	Yes	B	Nil	MC	B	B	Complete and incomplete	NA
40	PB12311	85%	66X	ALCL	TCRG, TCRB, TCRA	B	M	Nil	B	Nil	MC	Nil	M	Complete	Complete
41	226A	87%	69X	MEITL	TCRG, TCRB, TCRD	B	Nil	Yes	Nil	B	NA	NA	NA	NA	NA
42	z5469	88%	67X	MEITL	TCRG, TCRB, TCRA, TCRD	M	Nil	Yes	B	B	MC	M	B	Incomplete	Incomplete
43	z5340	88%	67X	MEITL	TCRG, TCRB, TCRA, TCRD	B	B	Nil	B	M	MC	M	B	Complete	Complete
44	T1473WK	89%	65X	ALCL	TCRG, TCRB, TCRA	B	M	Nil	B	Nil	NA	NA	NA	NA	NA
45	TR4251	20%	87X	ENKTL (T-origin)	TCRG, TCRB, TCRD	M	M	NA	Nil	M	NA	NA	NA	NA	NA
46	NKTL52	22%	77X	ENKTL (NK-origin)	None	Nil	Nil	NA	Nil	Nil	NA	NA	NA	NA	NA
47	T1479DS	41%	62X	ENKTL (T-origin)	None	Nil	Nil	NA	Nil	Nil	NA	NA	NA	NA	NA
48	TR4190	41%	90X	ENKTL (NK-origin)	None	Nil	Nil	NA	Nil	Nil	NA	NA	NA	NA	NA
49	FLN382	48%	60X	ENKTL (NK-origin)	None	Nil	Nil	NA	Nil	Nil	NA	NA	NA	NA	NA
50	NK-LJM	53%	62X	ENKTL (NK-origin)	None	Nil	Nil	NA	Nil	Nil	NA	NA	NA	NA	NA
51	76A	57%	82X	ENKTL (T-origin)	TCRG, TCRB	M	IND	NA	Nil	Nil	NA	NA	NA	NA	NA
52	ID1132	57%	75X	ENKTL (NK-origin)	None	Nil	Nil	NA	Nil	Nil	NA	NA	NA	NA	NA
53	ID1333	67%	81X	ENKTL (NK-origin)	None	Nil	Nil	NA	Nil	Nil	NA	NA	NA	NA	NA
54	ID1165	83%	85X	ENKTL (NK-origin)	None	Nil	Nil	NA	Nil	Nil	NA	NA	NA	NA	NA
55	HANK1	100%	40X	ENKTL-CL (NK-origin)	None	Nil	Nil	NA	Nil	Nil	NA	NA	NA	NA	NA
56	KAI-3	100%	40X	ENKTL-CL (NK-origin)	None	Nil	Nil	NA	Nil	Nil	NA	NA	NA	NA	NA
57	KHYG1	100%	40X	ENKTL-CL (NK-origin)	None	Nil	Nil	NA	Nil	Nil	NA	NA	NA	NA	NA
58	MEC04	100%	40X	ENKTL-CL (NK-origin)	None	Nil	Nil	NA	Nil	Nil	NA	NA	NA	NA	NA
59	NK92	100%	40X	ENKTL-CL (NK-origin)	None	Nil	Nil	NA	Nil	Nil	NA	NA	NA	NA	NA
60	NKYS	100%	40X	ENKTL-CL (NK-origin)	None	Nil	Nil	NA	Nil	Nil	NA	NA	NA	NA	NA
61	SNK1	100%	40X	ENKTL-CL (NK-origin)	None	Nil	Nil	NA	Nil	Nil	NA	NA	NA	NA	NA
62	SNK6	100%	40X	ENKTL-CL (NK-origin)	None	Nil	Nil	NA	Nil	Nil	NA	NA	NA	NA	NA
63	SNT8	100%	40X	ENKTL-CL (T-origin)	TCRG, TCRB	M	M	NA	Nil	Nil	NA	NA	NA	NA	NA
64	YT	100%	40X	ENKTL-CL (NK-origin)	None	Nil	Nil	NA	Nil	Nil	NA	NA	NA	NA	NA

Abbreviations: *TCR, T-cell receptor; BCR, B-cell receptor; WGS, whole-genome sequencing; TCRG, T-cell receptor gamma; TCRB, T-cell receptor beta; TCRA, T-cell receptor alpha; TCRD, T-cell receptor delta; PCR, polymerase chain reaction; AITL, angioimmunoblastic T-cell lymphoma; MEITL, monomorphic epitheliotropic intestinal T-cell lymphoma; PTCL, peripheral T-cell lymphoma; ENKTL, extranodal NK/T-cell lymphoma; ENKTL-CL, extranodal NK/T-cell lymphoma cell line; ALCL, anaplastic large cell lymphoma; HSTL, hepatosplenic T-cell lymphoma; IgH, immunoglobulin heavy chain; Nil, monoclonal rearrang ement not present; MC-monoclonal rearrangement; M, monoallelic monoclonal rearrangement; B, biallelic monoclonal rearrangement ; MC-IND, monoclonal rearrangement, unable to determine monoallelic vs biallelic rearrangement; IND, indeterminate; NA, not available; #small 1.1 kbp deletion present which cannot be reliably identified by segmentation algorithm*

Supplementary Table S2. BCR genes with monoclonal losses

Case no.	Case ID	Diagnosis	BCR genes with monclonal losses
1	PB000933	AITL	IgH
2	426A	AITL	IgH
3	z9951	AITL	NIL
4	z6425	PTCL	NIL
5	z3917	MEITL	NIL
6	AITL13	AITL	NIL
7	193A	PTCL	NIL
8	TR4254	AITL	IgH
9	z8343	PTCL	IgH
10	z5021	ALCL	NIL
11	z7889	PTCL	NIL
12	TR4323	AITL	NIL
13	TR4313	AITL	NIL
14	PB30818	ALCL	NIL
15	229A	AITL	IgH
16	PTCL02	PTCL	NIL
17	z7285	AITL	NIL
18	T1443KC	AITL	NIL
19	T1482TA	AITL	NIL
20	496A	AITL	NIL
21	TR4250	AITL	NIL
22	TR4241	AITL	IgH
23	69A	PTCL	NIL
24	211A	AITL	IgH
25	z4739	PTCL	NIL
26	z5286	ALCL	NIL
27	62A	PTCL	NIL
28	z3706	MEITL	NIL
29	TR4290	PTCL	NIL
30	AITL10	AITL	NIL
31	194A	AITL	NIL
32	SLS0697	HSTL	NIL
33	z2756	PTCL	NIL
34	z8043	AITL	NIL
35	TR4311	PTCL	NIL
36	z4110	ALCL	NIL
37	116A	AITL	NIL
38	TR4232	AITL	NIL
39	z3951	PTCL	IgL
40	PB12311	ALCL	NIL
41	226A	MEITL	NIL

42	z5469	MEITL	NIL
43	z5340	MEITL	NIL
44	T1473WK	ALCL	NIL
45	TR4251	ENKTL (T-origin)	NIL
46	NKTL52	ENKTL (NK-origin)	IgH
47	T1479DS	ENKTL (T-origin)	NIL
48	TR4190	ENKTL (NK-origin)	NIL
49	FLN382	ENKTL (NK-origin)	IgH
50	NK-LJM	ENKTL (NK-origin)	NIL
51	76A	ENKTL (T-origin)	NIL
52	ID1132	ENKTL (NK-origin)	IgH
53	ID1333	ENKTL (NK-origin)	NIL
54	ID1165	ENKTL (NK-origin)	NIL
55	HANK1	ENKTL-CL (NK-origin)	NIL
56	KAI-3	ENKTL-CL (NK-origin)	NIL
57	KHYG1	ENKTL-CL (NK-origin)	NIL
58	MEC04	ENKTL-CL (NK-origin)	NIL
59	NK92	ENKTL-CL (NK-origin)	IgH
60	NKYS	ENKTL-CL (NK-origin)	NIL
61	SNK1	ENKTL-CL (NK-origin)	NIL
62	SNK6	ENKTL-CL (NK-origin)	NIL
63	SNT8	ENKTL-CL (T-origin)	NIL
64	YT	ENKTL-CL (NK-origin)	NIL

Abbreviations: BCR, B-cell receptor; WGS, whole-genome sequencing; AITL, angioimmunoblastic T-cell lymphoma; MEITL, monomorphic epitheliotropic intestinal T-cell lymphoma; PTCL, peripheral T-cell lymphoma; ENKTL, extranodal NK/T-cell lymphoma; ENKTL-CL, extranodal NK/T-cell lymphoma cell line; ALCL, anaplastic large cell lymphoma; HSTL, hepatosplenic T-cell lymphoma; IgH, immunoglobulin heavy chain; IgK, immunoglobulin light chain kappa; IgL, immunoglobulin light chain lambda; Nil, monoclonal rearrangement not present.

Supplementary Table S3. Patterns of WGS TCR rearrangments by T-cell lymphoma subtype

	AITL (n=20)	ALCL (n=6)	HSTL (n=1)	MEITL (n=5)	PTCL (n=12)
NIL	0	1	0	0	0
TCRB	1	0	0	1	0
TCRG, TCRA	0	0	0	0	2
TCRG, TCRB	3	0	0	0	0
TCRG, TCRD	0	0	1	0	0
TCRG, TCRB, TCRA	16	4	0	0	8
TCRG, TCRA, TCRD	0	1	0	0	0
TCRG, TCRB, TCRD	0	0	0	1	0
TCRG, TCRB, TCRA, TCRD	0	0	0	3	2

Supplementary Table S4. Results of targeted sequencing of the T-cell receptor beta (TCRB) gene of selected T-cell lymphoma cases.

Case no.	Case ID	TCRB CNV status	Read count	% of clone	Junction sequences	Type	CDR3	V	D	J
37	116A	Loss	166670	16.7	TGCAGTGCTCGGGACTCCCCAGAGACCCAGTACTTC	Complete	CSARDSPETQYF	V20-1	D2	J2-5
	116A		641056	64.2	GACAGGGGGCACTGAAGCTT	Incomplete			D1	J1-1
31	194A	Loss	713760	73.3	GGGACGGTGGATCCGAGCAGTAC	Incomplete			D1	J2-7
24	211A	Loss	464159	25	TGTGCCAGCAGTCCGGGGACAGGGGCGACCTACGAGCAGTACTTC	Complete	CASSPGTGATYEQYF	V6-5	D1	J2-7
	211A		554341	29.9	CATTGTGGGGTTAACTATGGC	Incomplete			D1	J1-2
23	69A	Loss	904804	54.9	TGTGCCAGCAGCCAACACGAAGACCCCTACGAGCAGTACTTC	Complete	CASSQHEDPYEQYF	V3-1		J2-7
42	Z5469	Loss	783334	50.1	TGTGGGGACTGTTCCTACGAGC	Incomplete			D2	J2-7
	Z5469		514228	32.9	TGTGGGGACACCCCTCTAACTATGG	Incomplete			D1	J1-2
32	SLS0697	No loss	531215	78.9	GGGACAGGGGTCCGGAAACA	Incomplete			D1	J1-3
12	TR4323	Loss	304912	34.6	TGCGCCAGCAGCTTGGTAGGGGAAGACAATCAGCCCCAGCATTTT	Complete	CASSLVGEDNQPQHF	V5-1	D1	J1-5
28	Z3706	Loss	86952	60.8	GGGGACAGGGCTTATCAAGAGACCC	Incomplete			D1	J2-5
26	Z5286	No loss	188152	30.6	GGGACAGGGGCTGCGTGTGGGTAACACCA	Incomplete			D1	J1-3
43	Z5340	Loss	1163640	58	TGTGCCAGCAGCGCCGGACTAAACAATGAGCAGTTCTTC	Complete	CASSAGLNNEQFF	V2	D2	J2-1
	Z5340		821759	41	TGTGCCAGCAGCGTAGCGCGGCGGCTATACAATGAGCAGTTCTTC	Complete	CASSVARRLYNEQFF	V9		J2-1
40	PB12311	Loss	1414255	90.8	TGTGCCAGCAGTTACAAGAGGAGACAGGGGTCTTCACCCCTCCACTTT	Complete	CASSYKRRQGSSPLHF	V6-5	D1	J1-6
16	PTCL02	No loss	488237	29.9	TGTGCCAGCAGTGAAGGACAGGGCAATCAGCCCCAGCATTTT	Complete	CASSEGQGNQPQHF	V6-1	D1	J1-5
	PTCL02		482655	29.6	TGGGGACAGGAACACTGAAG	Incomplete			D1	J1-1

Supplementary Table S5. Copy number variation data of B cell receptor genes

Accession ID	**Name of cell line**	Diagnosis	No. of IG genes showing monoclonal losses	IG genes with monoclonal losses	TCR genes with monoclonal losses
SRR1236466	OCI-Ly1[P8]	DLBCL	3	IgH, IgK, IgL	Nil
SRR1236467	NU-DHL-1[P11]	DLBCL	3	IgH, IgK, IgL	Nil
SRR1236469	NU-DUL-1[P9]	DLBCL	3	IgH, IgK, IgL	TCRG
SRR1236470	OCI-Ly7[P8]	DLBCL	3	IgH, IgK, IgL	Nil
SRR1236471	OCI-Ly3[P10]	DLBCL	3	IgH, IgK, IgL	Nil
SRR1236472	MD903[P7]	DLBCL	3	IgH, IgK, IgL	Nil
SRR1236473	SU-DHL-9[P7]	DLBCL	3	IgH, IgK, IgL	Nil
SRR1236475	WSU-DLCL2[P6]	DLBCL	3	IgH, IgK, IgL	Nil
SRR1236476	SU-DHL-6[P8]	DLBCL	2	IgK, IgL	Nil
SRR1236477	OCI-Ly19[P9]	DLBCL	3	IgH, IgK, IgL	Nil

Abbreviations: DLBCL, diffuse large B-cell lymphoma; IG, immunoglobulin; TCR, T-cell receptor; IgH, immunoglobulin heavy chain; IgK, immunoglobulin light chain kappa; IgL, immunoglobulin light chain lambda.
